# Capturing Ecosystem Services, Stakeholders' Preferences and Trade-Offs in Coastal Aquaculture Decisions: A Bayesian Belief Network Application

**DOI:** 10.1371/journal.pone.0075956

**Published:** 2013-10-14

**Authors:** Laetitia Helene Marie Schmitt, Cecile Brugere

**Affiliations:** 1 Centre for Health Economics/Department of Economics and Related Studies, University of York, Heslington, York, United Kingdom; 2 Stockholm Environment Institute, University of York, Heslington, York, United Kingdom; Catalan Institute for Water Research (ICRA), Spain

## Abstract

Aquaculture activities are embedded in complex social-ecological systems. However, aquaculture development decisions have tended to be driven by revenue generation, failing to account for interactions with the environment and the full value of the benefits derived from services provided by local ecosystems. Trade-offs resulting from changes in ecosystem services provision and associated impacts on livelihoods are also often overlooked. This paper proposes an innovative application of Bayesian belief networks - influence diagrams - as a decision support system for mediating trade-offs arising from the development of shrimp aquaculture in Thailand. Senior experts were consulted (n = 12) and primary farm data on the economics of shrimp farming (n = 20) were collected alongside secondary information on ecosystem services, in order to construct and populate the network. Trade-offs were quantitatively assessed through the generation of a probabilistic impact matrix. This matrix captures nonlinearity and uncertainty and describes the relative performance and impacts of shrimp farming management scenarios on local livelihoods. It also incorporates export revenues and provision and value of ecosystem services such as coastal protection and biodiversity. This research shows that Bayesian belief modeling can support complex decision-making on pathways for sustainable coastal aquaculture development and thus contributes to the debate on the role of aquaculture in social-ecological resilience and economic development.

## Introduction

The shrimp aquaculture industry of Thailand, which accounted for 15.4% of the global aquaculture production of shrimps in 2009 [1], is a major contributor to the country's economy, bringing in over US$ 2 billion of foreign revenues annually [2] and employing more than one million people [3]. The majority of the industry (98%) is based on the production of the exotic Pacific white-leg shrimp (*Litopenaeus vannamei*), which has replaced the indigenous Giant black tiger shrimp (*Penaeus monodon*) since the early 2000s [4].

The introduction of intensive farming techniques in the 1980s led to Thailand becoming a world-leading shrimp producer. However, this shift was associated with water pollution and acidity build-up in shrimp ponds. In conjunction with poor husbandry methods, this resulted in the onset of catastrophic viral diseases and a collapse in production [5], [6], prompting farmers to abandon their ponds and dig new ones further into mangrove-forested areas. Mangroves swamps were considered of low economic value as the ecosystem services they deliver (e.g. pollutant sink, storm protection, provision of wood and fish [7], [8]) were not taken into account. As a result, Thailand's mangrove forests were halved between 1961 and 1993, although the extent to which shrimp farming alone was responsible for the loss of mangrove cover in the country remains debatable [5].

The Ecosystem Approach to Aquaculture (EAA) has recently emerged in response to calls for increased sustainability in the aquaculture sector and recognition that aquaculture is often embedded in sensitive social-ecological systems [9]. This approach advocates the consideration of biophysical and human dimensions of ecosystems in order to achieve the dual aims of sustainable production and human wellbeing [10]. At the heart of the EAA lies the notion of social-ecological resilience, defined as “the capacity to maintain integrity when responding to external changes and feedbacks” [11]. Decision-making in the context of coastal social-ecological systems requires considering several different issues: i) interconnectivity, i.e. the fact that “disruption in one system is likely to cause disruption in the other” [10], (ii) complexity and uncertainty, as coastal ecosystem responses are often non-linear [12] and the minimum level of ecosystem structure needed to maintain a constant flow of services is unknown [13], and (iii) stakeholder conflicts stemming from competing uses over coastal resources and institutional failures [14].

However, a gap in the methodology exists as these issues are not simultaneously considered in decision-making or when the EAA is implemented in coastal areas. This calls for a decision support system that can holistically assess and quantify the trade-offs arising from coastal aquaculture management decisions. A prior review of the range and uses of decision support tools currently available (e.g. extended cost-benefit analysis, multi-criteria decision analysis) showed that Bayesian Belief Networks (BBN) – also called influence diagrams – stand out for their ability to handle uncertainty, complex non-linear relationships as well as qualitative and quantitative data of an ecological and economic nature. BBNs also have the advantage of remaining straightforward enough to allow clear communication of complex decision outcomes to policy makers [15].

BBNs have been applied to natural resource management to model human pressures on diverse ecosystems [16]–[18], to assist in the assessment of the social, ecological and economic dimensions of coastal water and catchment management [19]–[21] and to evaluate and compare fisheries management plans [22]. Applications of BBNs to aquaculture have been limited to modeling the impacts of water management options on the simultaneous production of rice, fish, crab and shrimp in Viet Nam [23] and to quantifying the human drivers behind the adoption of sea cucumber aquaculture as an alternative livelihood strategy in Tanzania [15].

The purpose of the present study, and the novelty of this research, is to broaden the use of Bayesian Belief Networks by incorporating indicators of social-ecological resilience and refining their application to aquaculture. The specific objective of this work is to show how such a decision support tool can help mediate complex and multiple trade-offs in the context of shrimp aquaculture development in Thailand through: (i) the articulation of available knowledge on the impacts of coastal aquaculture on social, ecological and economic systems via a network structure, and (ii) the generation of a probabilistic impact matrix that highlights trade-offs – or “social conflicts over interest and values” [24] – and uncertainties in management decision outcomes. The underlying goal is to provide policy-makers with a full picture of the potential consequences of their aquaculture development decisions on specific characteristics of the system. Section 2 of this paper presents the methodology used to develop a BBN applied to shrimp aquaculture. Section 3 shows modeling results for six scenarios of aquaculture management and assesses their implications in terms of trade-offs between ecosystem services provision, local livelihoods, financial profits and economic development. Finally, section 4 discusses the findings and the role of BBN as a decision-support system for sustainable aquaculture development, in line with the EAA.

## Materials and Methods

### 1. Study area and data collection

In Thailand, the shrimp farming sector is characterized by the prevalence of small production units (<2 ha), which coexist alongside a minority of very large farms [25]. As of 2007, more than 90% of shrimp farms were intensive, i.e. with high stocking densities of juvenile shrimp in ponds [26]. Production predominantly takes place in semi-closed systems characterized by minimal exchange of water with the outside environment to limit the introduction of viruses and pathogens and the release of pollution [27]–[29]. However, pond water and sludge are still partially discharged in adjacent waterways, notably at the time of harvest. In addition, *L. vannamei* shrimps have been reported in the vicinity of ponds, despite measures aimed at preventing their accidental release in the environment [30]. Fully closed systems also exist but their requirements of a water reservoir, sedimentation pond and continuous aeration make them capital-intensive operations [28]. Whilst shrimp aquaculture has increasingly taken place in supra-tidal areas, i.e. behind the mangrove fringe (S. Funge-Smith, personal communication, 2011), a large number of shrimp farms are still suspected to be operating in inter-tidal areas due to lack of law enforcement and legislation loopholes [5], [31].

Nevertheless, awareness of Better Management Practices (BMPs), framed by the International Principles for Responsible Shrimp Farming [32], has been growing. BMPs aspire to reduce the impact of shrimp farming on the environment and improve the livelihoods of farmers by implementing measures such as storage ponds and increasing efficiency in production by optimizing the shrimp feeding process [33]. In Thailand, the implementation of BMPs has been promoted, in particular among small producers (e.g. [34]), but their adoption remains limited in comparison to other countries such as India and Viet Nam [35], [36].

At the other end of the production spectrum is aquasilviculture, an extensive system that promotes harmonious aquatic production alongside mangrove forestation [37], [38]. In the context of the present research, aquasilviculture is defined as a mangrove-shrimp system where 70% of the pond surface is planted with mangroves and 30% is dedicated to shrimp production. Although aquasilviculture remains marginal in Thailand in comparison to other Southeast Asian countries, it has enabled farmers to return to traditional low input-low output shrimp farming after crop failures [39]. It is also recognised that, under the right conditions, it can be used as a suitable management strategy to rehabilitate disused shrimp ponds, enhance the provision of mangrove ecosystem services, and provide a complementary source of income to farmers [40], [41].

**Figure 1:**
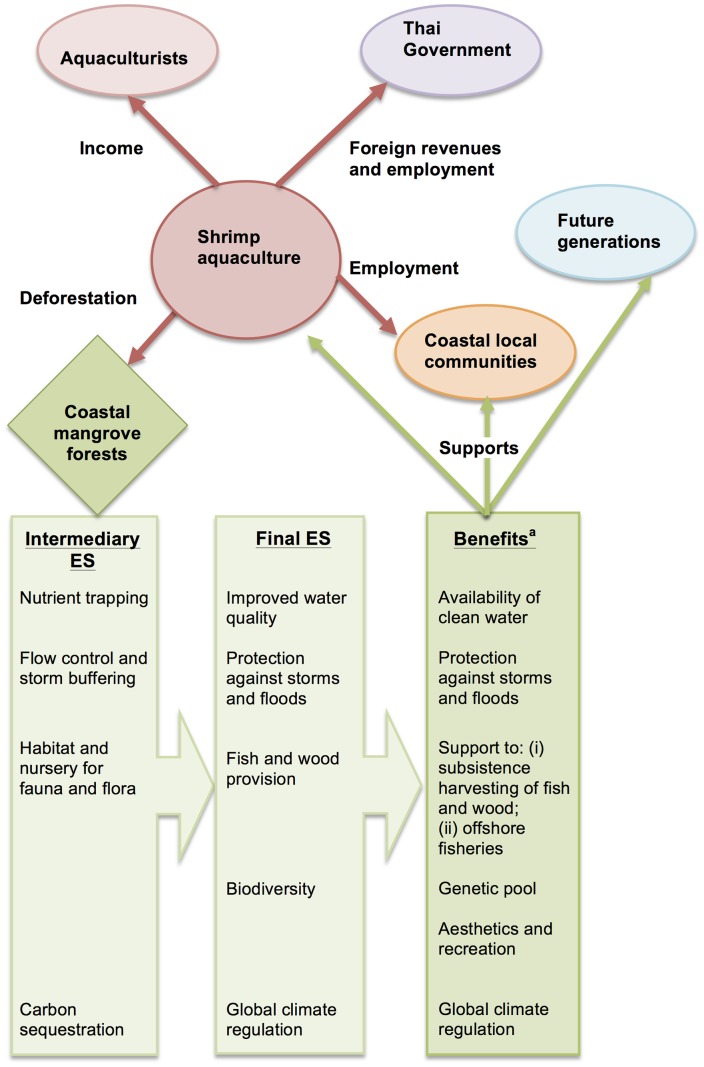
Aquaculture's main impacts on the environment, the economy and livelihoods and related user-conflicts. ES = Ecosystem Services. ^a^ Benefits should be distinguished from final ES since they are often a product of final ES and human inputs (e.g. fishing gear) and economic valuation of ecosystem should apply to ecosystem benefits only [13], [54], [55]. Figure based on information from [40] and [8], relying on the ecosystem services classification by [13], [55].

The characteristics of the development of shrimp farming in Thailand, its history and impacts have been extensively documented and general information and data on the sector were collected from the literature. In line with the EAA, Figure 1 captures the multiple interactions of the sector with the environment (including services provided by coastal ecosystems), the economy and livelihoods. Four groups of key stakeholders have been identified (underlined in Figure 1): (i) farmers themselves, organized in associations and seeking income from their activity, (ii) the Government of Thailand, seeking export revenues and employment through the entire shrimp value chain, (iii) members of coastal communities who can simultaneously benefit (e.g. employment) and suffer (e.g. loss of mangrove-dependent livelihoods) from the conversion of mangroves to ponds, and (iv) future generations, representing the stakes of long-term sustainable coastal development.

**Figure 2:**
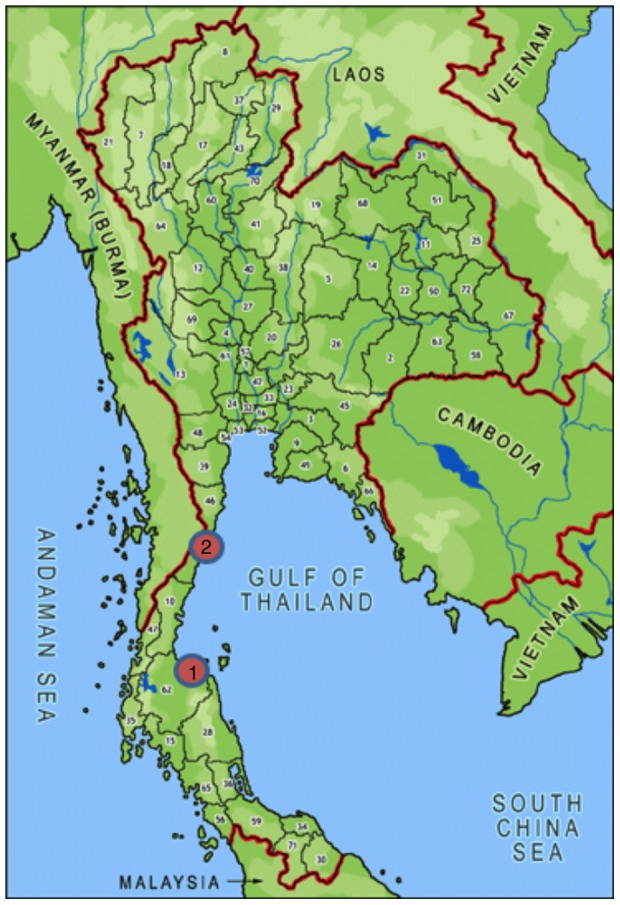
Locations of onsite surveys in Thailand. 1: Surat Thani (Surat Thani Province) representing conventional shrimp farming. 2: Samroyiot (Prachap Kiri Khan Province) representing small-scale shrimp farming.

The bulk of the literature on the economics of shrimp farming relates to *P. monodon* and not *L. vannamei*, which is associated with different yields, costs and profits [4]. To fill this gap and provide up-to-date data on exploitation costs and revenues for *L. vannam*ei, onsite surveys were implemented. Ten intensive shrimp farmers were sampled in Surat Thani, Surat Thani Province (sample stratified by farm size: 3 very small farms (<2 ha), 3 small farms (2.1–5 ha), 3 medium farms (5–50 ha) and 1 large farm (>50 ha)). Ten smaller-scale intensive farmers were also sampled in Samroyiot, Prachuap Kiri Khan Province (Figure 2). Of the Samroyiot farmers, six were implementing Better Management Practices (BMPs) and also provided economic and production data. These six farmers were gathered in a “cluster” and working together towards the implementation of BMPs.

Ethical clearance of the research was provided by the European Commission under Framework Programme 7 (PIEF-GA-2009- 235835) as a condition of the award. Questionnaires were designed and tested to ensure an optimal balance between cultural sensitivity and achievement of the study objectives. Particular attention was given to gender and to the sensitiveness of the questions asked when interviewing women, minority or vulnerable people (e.g. elders, disabled, illiterate, poor), in order to ensure the free expression of their views. Informed verbal consent was provided by each interviewee in light of guarantees of confidentiality, anonymity, privacy, data protection and the possibility to withdraw from the survey at any time. Ethical training was provided to all partners involved in data collection. Monitoring and evaluation of participation and of the research process occurred according to agreed codes of conduct and standards of research practice, including anonymizing the questionnaire recording forms and destroying these at the end of the study.

### 2. BBN development

#### 2.1. BBN definition

A BBN is a directed acyclic graph, which parameters are treated as random variables and modeled via nodes. The typology of nodes used in BBN modeling is provided in Table 1. Nodes are interconnected in “parent-child” (i.e. predictive) relationships. A “child” node has a conditional probability distribution (CPD) for each possible combination of states of its “parent” nodes and the width of the distributions indicates the level of uncertainty in the cause-effect relation [42]. Unlike hierarchical Bayesian networks, which rely extensively on model simulations, the CPDs that populate BBN chance nodes are generally obtained analytically, with expert opinion used to overcome data gaps [43]. Scenario analysis allows the updating of chance nodes' CPDs into posterior probability distributions (PDs) via Bayes' rule of joint probabilities [44].

**Table 1:** Typology of nodes in BBN modeling, according to their modeling role and position in the network.

**Modeling role**	
**Chance node**	Node modeling a random variable over discrete states and defined by a joint conditional probability distribution
**Utility node**	Node populated with utility values that express preferences over outcomes
**Decision node**	Node modeling choices that can be made by the decision maker, comprising one state for each scenario
**Position in network**	
Parent node	Node predicting one (or more) child node(s)
Child node	Node linked to one (or more) parent node(s) via a predictive relationship. A child node has a conditional probability distribution for each combination of state of its parent nodes
Root node	A node with no parent, defined by a probability distribution
Leaf node	A node with no child

**Figure 3:**
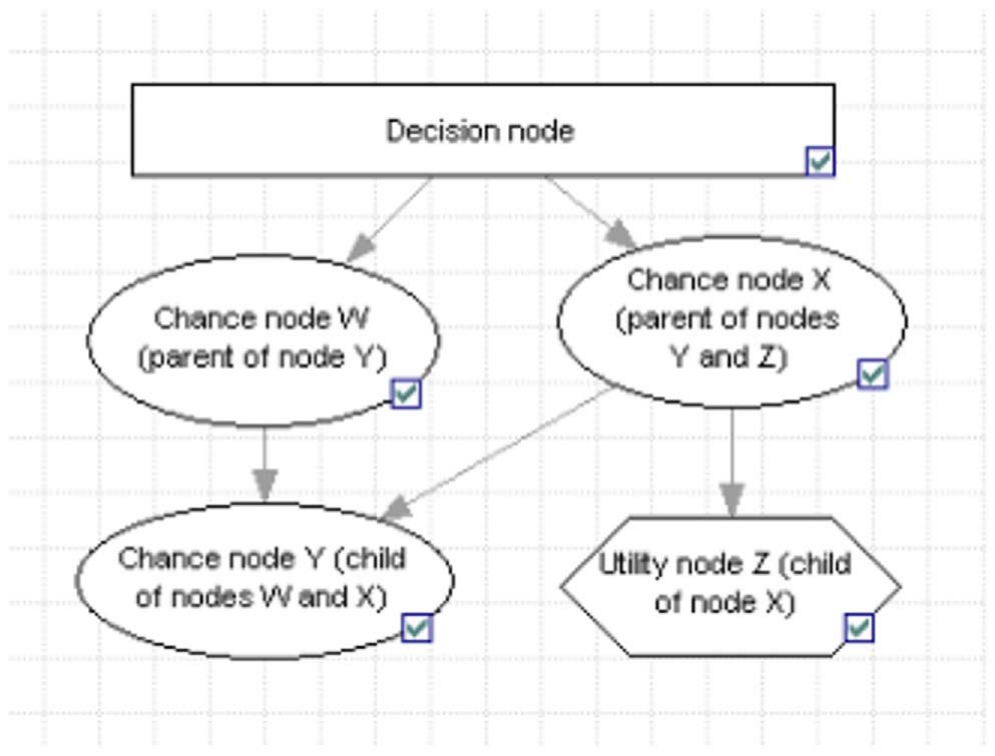
Convention adopted to graphically represent each type of node in a Bayesian Belief Network.

In addition to explicitly handling uncertainty via the notion of conditional probabilistic dependence between variables [45], a BBN can incorporate both qualitative and quantitative information by connecting a decision node with chance nodes and utility nodes (Figure 3). Finally, thanks to its network structure, a BBN provides a visual representation of the causal relationships that underpin complex systems to which management decisions are applied, as well as straightforward probabilistic information on uncertain outcomes of management actions [46], [47].

#### 2.2. Network construction

Information on the impacts of shrimp farming collected from the literature was incorporated in a network via chance nodes, whereby cause-effect relationships were made explicit. Leaf nodes, which are the nodes of focus for our scenario analysis, were specifically chosen to represent the diversity of interests of the four groups of stakeholders previously identified, so that user-conflicts could be made explicit in output results. The BBN was developed via the software GeNIe from the Decision Systems Laboratory of the University of Pittsburgh, USA (http://dsl.sis.pitt.edu). This software is freely available and has the advantage over other free commonly used software of not limiting the network size.

Following Marcot et al.'s guidance [48], the number of parent nodes per child node was limited to three so that the number of CPDs of child nodes would remain within reasonable limits. As a consequence, three modeling choices were made:

- The BBN was built at the shrimp farm level and thus did not model the whole shrimp value chain. Nevertheless, employment generated by the sector was indirectly dealt with via the node “long-term contribution to the country's shrimp exports”.
- The issue of abandoned ponds was addressed in the model via the node “long term contribution to the country's shrimp exports” (Thai government's perspective) and the computation of the net present value (NPV) of the profit earned by aquaculturists.
- The modeling of coastal communities' resilience, which was inspired by Ashley et al.'s livelihoods framework [49], focused solely on natural, human and financial capital, which were the most straightforward to handle in the present analysis. Modeling of these three sources of capital was made via the nodes “fish/wood for locals”, “health of locals” and “potential for income diversification” respectively.

The modeling of mangroves' ecosystem services called upon a value judgment on how the value of these services would be best communicated. Mangroves' ecosystem services were all modeled qualitatively via chance nodes. However, coastal protection was also modeled quantitatively in monetary units (utility node) as it is estimated to be mangroves' most economically valuable service [12].

#### 2.3. Case study and management scenarios

A hypothetical case study representing the range of pond management possibilities was defined to show how the constructed BBN could support complex decision-making on coastal aquaculture development pathways. This case study used six mutually exclusive aquaculture and land management scenarios (Table 2), underpinned by a set of assumptions defined in Table 3.

**Table 2:** Mutually exclusive aquaculture and land management scenarios used in BBN modeling.

*Scenario name*	*Description*
BAU - Business as usual	Conventional intensive farming as currently practised
BMP	Introduce Better Management Practices
Restore forest+closed-system	Fully restore the existing farm as a mangrove forest and build a closed-system in the supra-tidal zone, behind the mangrove fringe
Replant 20%	Replant mangroves on 20% of the farm pond area
Replant 40%	Replant mangroves on 40% of the farm pond area
Aquasilviculture	Replant mangroves on 70% of the pond area and integrate the culture of mangroves with low-density shrimp aquaculture

*Notes*:

- The last three scenarios combine intertidal land conversion with mangroves replanting and conservation and were designed to capture non-linearity in mangroves provision of ecosystem services [12].

- The “aquasilviculture” and “restore forest+closed-system” scenarios were associated with a 100% probability of “high” level of coastal protection, while “BAU” and “BMP” scenarios were associated with a 100% probability of “low” level of coastal protection.

**Table 3:** Assumptions behind the BBN modeling case study.

*Assumption*	*Description*
A1	Small-scale intensive shrimp farms relying on semi-closed systems are operating as a group and cultivate and export the non-native species *L. vannamei* (using specific pathogen free broodstock), thus generating cumulative impacts on the environment
A2	Farms are located in Thailand's intertidal area (i.e. formerly forested by mangroves), and have recently started operating
A3	The farm owner fully undertakes the management option of his/her choice at t = 0(1)
A4	The rate of survival of replanted mangroves is high and replanted mangroves provide their ecosystem service straightaway(1) (2)
A5	All production, including from aquasilviculture, is exported(3)
A6	To the exception of aquasilviculture, stocking densities remain identical among the different management scenarios

*Notes*:

(1) A3 and A4 were motivated by the difficulty to integrate time dynamics in BBNs.

(2) Mangroves are expected to deliver their services after 3 years of normal growth (J. Primavera, personal communication, 2011).

(3) While the quality and size of shrimps may greatly determine whether shrimps are exported or not, this assumption reflects the fact that 85% of the Thai shrimp production is exported [3].

#### 2.4. Expert involvement

A group of 42 senior experts in coastal aquaculture from academia, research institutions and the industry were contacted by email to validate the network and the case study and to elicit the CPDs that populate chance nodes. They were sent conditional probability tables to fill for each chance node, alongside extensive explanations about the constructed BBN, the case study and the management scenarios considered. Of these experts, twelve contributed to the fine-tuning of the network variables and causal relationships, and to the refinement of the assumptions underpinning the case study. CPDs were elicited by four of the twelve experts through a number of iterations and an in-depth dialogue.

#### 2.5. BBN parameterisation

*Chance nodes.* Because biophysical modeling was outside the scope of this research and due to lack of available datasets, all the CPDs of the model's chance nodes were elicited by experts. As the BBN comprises a large number of chance nodes (24), all chance nodes were attributed the states of “high”, “medium” and “low” to facilitate the CPD elicitation exercise. Although applying such a restriction on the states of variables clearly represents a simplification of reality, it ultimately enables information on trade-offs to be presented in an easily understandable form to decision-makers. Normalized average values of elicited CPDs were then calculated.

**Figure 4:**
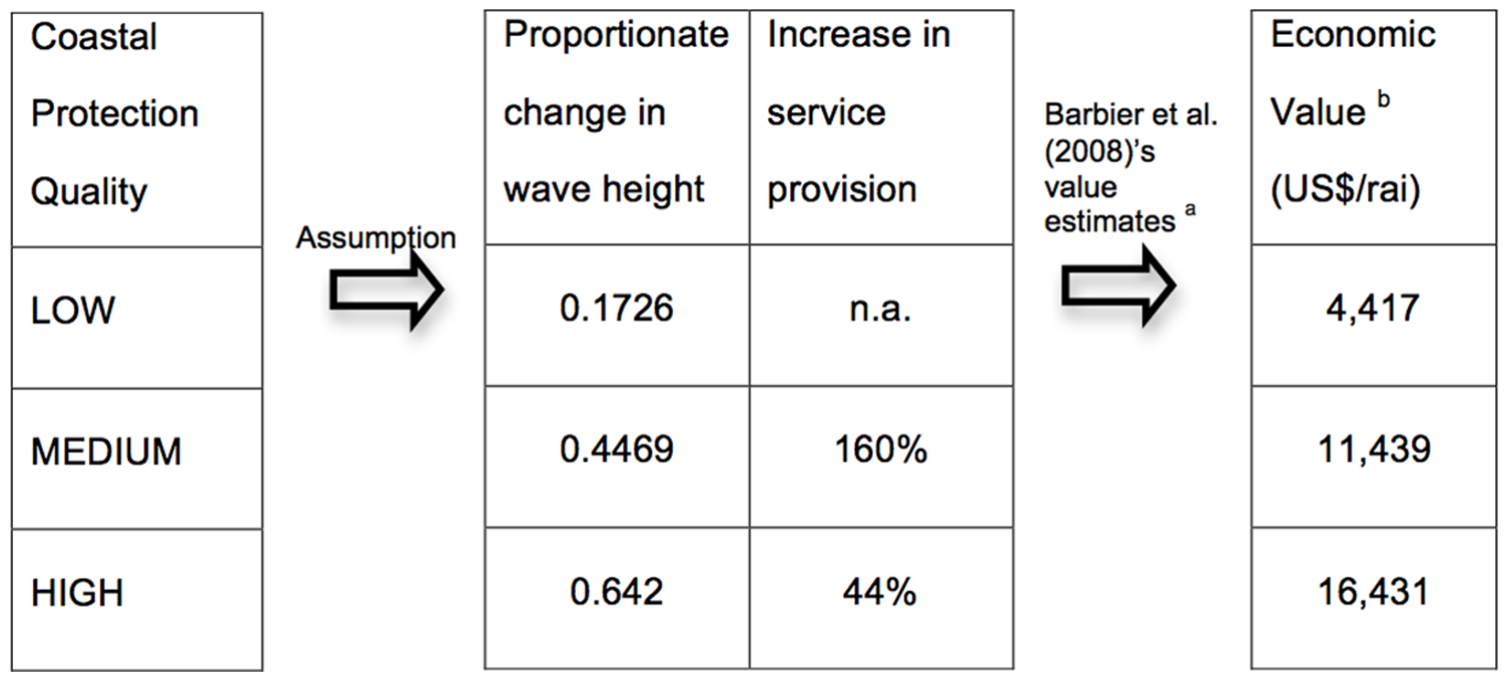
Parametrisation of the utility node “Value of coastal protection”. ^a^ Estimates computed by [12] based on the expected damage cost method combined with a quadratic wave attenuation function and the assumption that each km^2^ of mangroves represents a mangrove forest area of 100 m inshore along a 10 km coastline. Estimates were converted in (US$/rai) with 1 rai = 0.0016 km^2^. ^b^ Net present values computed over a 20-year time-horizon with a 10% discount rate.

*Utility node for coastal protection.* Barbier at al. found that mangroves' level of wave attenuation was a quadratic function of habitat size, which allowed computation of a set of estimates for the monetary value of coastal protection services [12]. In order to link these estimates to the qualitative level of coastal protection quality modeled in the BBN, each coastal protection level (“high”, “middle”, “low”) was arbitrarily associated to a non-linear change in wave height and the monetary value estimates were rescaled to rai units. This procedure, which enabled coastal protection values to be integrated into the model, is summarized in Figure 4.

*Utility nodes for aquaculturists' annual profit.* The computation of the profit earned by the group of aquaculturists encompassed only the costs and revenues expected to be substantially impacted by the type of farm management. Therefore, the term “profit” here is an indicator of the financial performance of each management option, and not of the accounting profit earned. Annual profit was expressed in US$/rai so that it could be easily rescaled to any number of farms. Table S1, in supporting information, details how economic and production data from onsite surveys in Surat Thani and Samroyiot were integrated in the profit computations, in combination with secondary data and expert opinion.

For each management scenario*s*, aquaculturists' annual profit was computed in GeNIe, via several utility nodes, based on formulas (1) and (2). The net present value (NPV) was computed in Excel according to formula (3).
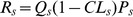
(1)

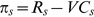
(2)

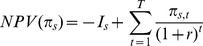
(3)

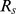
 is aquaculturists' annual gross revenue (US$/rai, 1 rai = 0.16 ha); 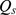 is the total annual shrimp production (kg/rai); 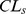 is the annual crop loss (%); 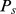 is the shrimp farmgate price (US$/kg); 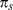 is the annual profit (US$/rai); 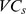 is the annual production variable cost (US$/rai); 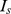 is the investment cost (US$/rai); 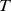 is the farm lifespan (years) and 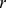 is the discount rate.

## Results

### 1. Network and impact matrix

**Figure 5:**
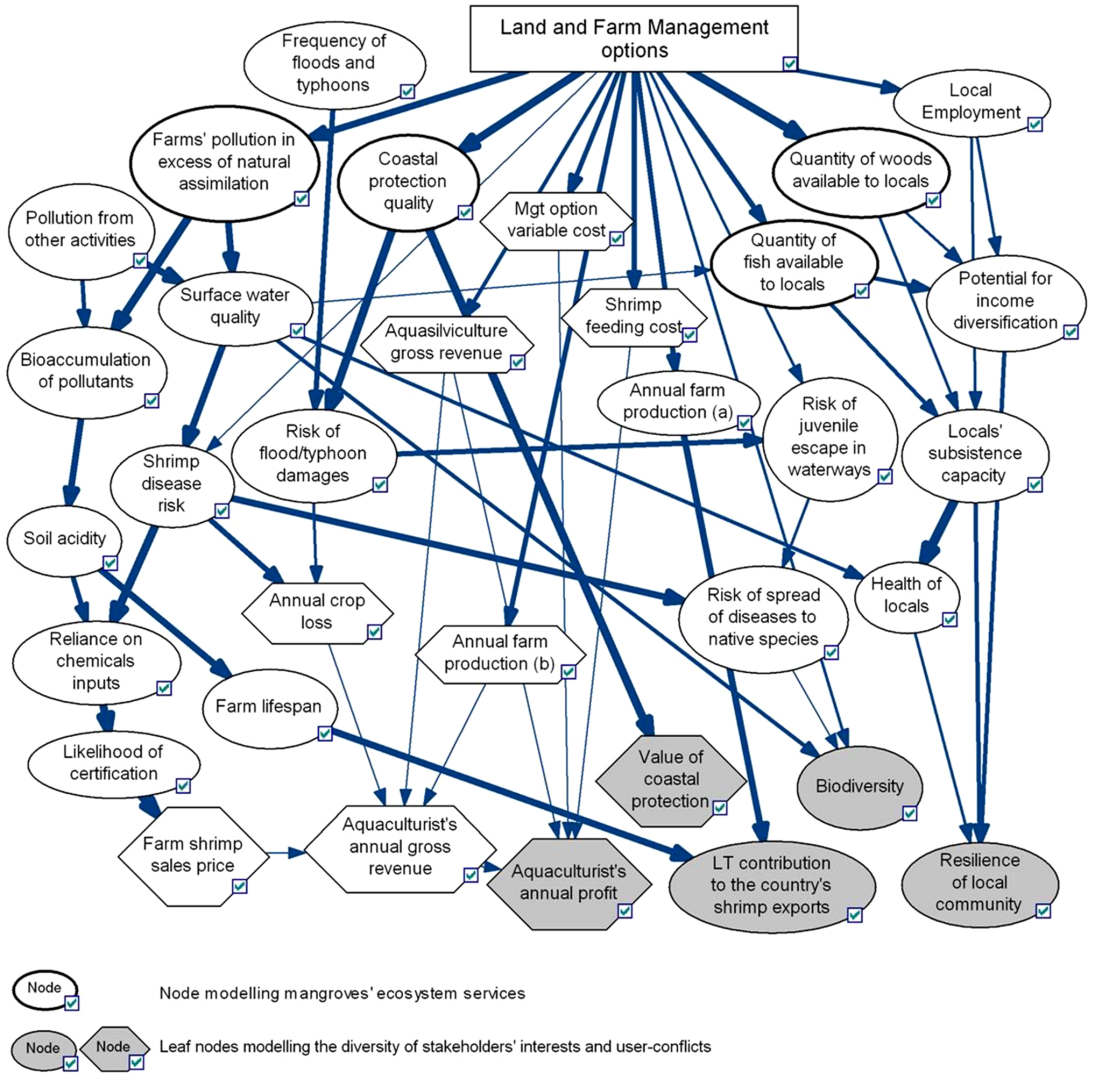
Bayesian belief network and strength of influence between variables. The width of the arrows linking nodes depicts the strength of influence between parameters ciphered by the conditional probability distributions. ^(a)^ Chance node defining farm production qualitatively over the states “high”, “medium” and low”. ^(b)^ Utility node defining farm production quantitatively (kg/rai).

**Figure 6:**
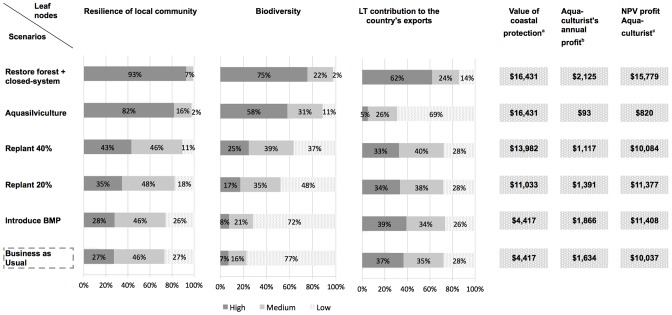
Impact matrix. Following belief propagation, posterior probability distributions and expected utility values for each leaf node were obtained for each management scenario. The matrix summarizes the performance of the five management scenarios against the BAU scenario on the criteria modeled via the leaf nodes. ^a^ NPV values (US$/rai) computed over a 20 year-time horizon, using a 10% discount rate. ^b^ Encompasses only exploitation costs and revenues impacted upon by the management option. ^c^ NPV values (US$/rai) computed using a 10% discount rate and based on estimates of farm lifespan (see Table S1). 1 ha = 6.25 rai.

The BBN in Figure 5 provides a graphical representation of the knowledge and belief of causal relationships between social, ecological and economic impacts associated with land and farm management scenarios relative to shrimp farming. Modeling output for the five leaf nodes (aquaculturists' annual profit; long-term contribution to the country's shrimp exports; value of coastal protection; biodiversity; resilience of local community), which were specifically chosen to reflect trade-offs and user-conflicts, are presented in the form of a probabilistic impact matrix (Figure 6). The scenario “restore forest+closed-system” scored the highest on each criterion modeled via the leaf nodes and thus represents the best-case scenario.

### 2. Interpretation and sensitivity analyses

#### 2.1 Profit

Whilst levels of farm production drove the difference in annual profit between management scenarios, production loss due to mangrove replanting in ponds was partially compensated by a lower risk of shrimp loss from disease outbreak or typhoons and a higher likelihood of securing a price premium via production certification. For instance, the annual profit under scenarios “replant 20%” and “replant 40%” was lower than under “BAU” by only 15% and 32% respectively. This was due to a lower expected crop loss (10.3% and 8% of annual crop respectively, vs. 12.6% for “BAU”) and a greater likelihood of certification (12.7% and 19.7% respectively, vs. 8% for “BAU”). The better financial performance of scenario “BMP” against “BAU” stemmed from lower feeding costs following optimization of the shrimp feeding process.

Sensitivity to assumptions underpinning the NPV computations was tested. As a result of compounded discounting, sensitivity to discount rate was especially acute for scenarios “aquasilviculture” and “restore forest+closed-system”, since these management systems were assumed to be operating over the longest periods (50 years and 30 years respectively). Conversely, sensitivity to farm lifespan was stronger for scenarios “BAU” and “BMP” since they were associated with a shorter lifespan (10 and 13 years respectively). Under the assumption that all systems would operate for only 10 years, scenario “restore forest+closed-system” was no longer associated with the greatest NPV of profits due to the high investment cost incurred in year 0 (US$ 4,249/rai).

#### 2.2 Coastal protection value

The larger the replanted pond surface was, the higher the expected value of coastal protection. Following assumptions pertaining to the probability of coastal protection levels associated with each management scenario (see Table 2), the BBN encompassed uncertainty about coastal protection quality only for the scenarios which involved replanting 20% and 40% of the pond surface. Economic values of coastal protection were underpinned by the assumed association between qualitative levels of protection and quantitative provision of wave-attenuation service (see Figure 3). Sensitivity analysis showed that the stronger the assumption of non-linearity, the more spread out across management scenarios coastal protection values were.

#### 2.3 Long-term contribution to the country's exports

The performance of scenarios “aquasilviculture” and “restore forest+closed-system” on the long-term contribution to the country's exports differed sharply from the “BAU” scenario. It was expected to be high with a 62% probability under scenario “restore forest+closed-system” (vs. 37% under “BAU”), while it was expected to be low with a 69% probability under scenario “aquasilviculture” (vs. 28% under “BAU”). By contrast, in the other scenarios, the level of contribution to exports did not differ substantially from the “BAU” scenario as the decrease in production due to replanting was partially offset by an increase in farm lifespan (lower bioaccumulation of pollutants). For example, the likelihood of high contribution to exports under scenario “replant 40%” was only 4 percentage points lower than under “BAU”.

#### 2.4 Biodiversity

**Figure 7:**
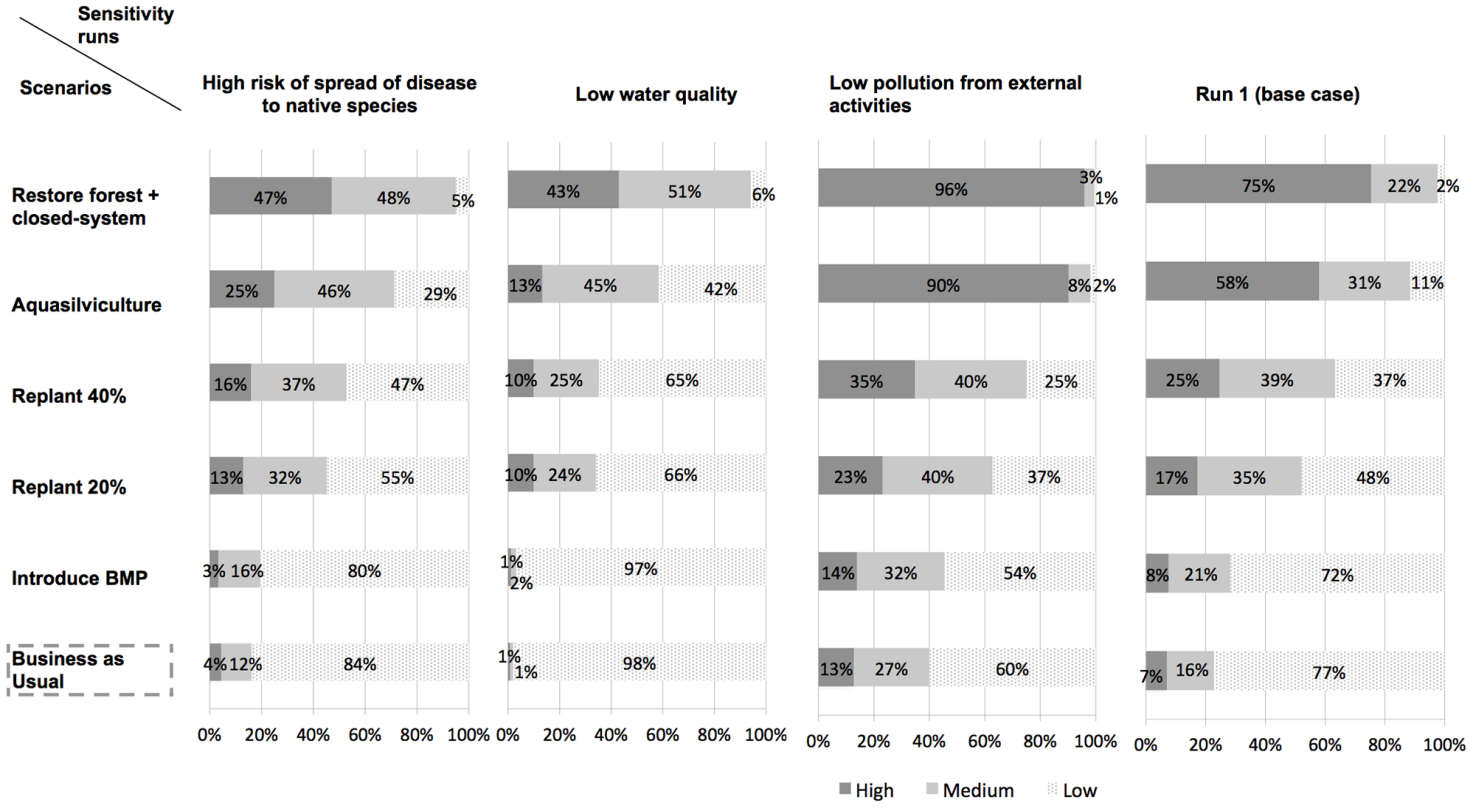
Sensitivity analyses for the node “Biodiversity”. Assessment of the predictive influence of the variables “risk of spread of diseases to native species”, “surface water quality” and “pollution from other activities” on the posterior probability distribution of the node “biodiversity”.

Whilst biodiversity levels (“high”, “medium”, “low”) were not specifically defined here, they were meant to reflect the degree of variety of living organisms and genetic variability encapsulated in the definition of biodiversity adopted in Article 2 of the Convention on Biological Diversity in 1993. The likelihood of a high level of biodiversity was shown to increase from 7% under “BAU” to 8% under “BMP”, 17% under “replant 20%”, 25% under “replant 40%”, 58% under “aquasilviculture” and 75% under “restore forest+closed-system”. Interestingly, the likelihood of high biodiversity increased by 50% between scenarios “replant 20%” and “replant 40%” and by 130% between scenarios “replant 40%” and “aquasilviculture” (i.e. replant 70%), highlighting non-linearity in ecosystem service provision. Sensitivity analysis consisted of evaluating the separate impacts on biodiversity levels under each sensitivity scenario of: (i) a high risk of spread of diseases to native species; (ii) high water quality and (iii) low pollution from other activities, against the base case simulation (run 1) where the root node “pollution from other activities” was populated with a uniform PD. For these sensitivity analyses, the nodes “risk of spread of diseases to native species”, “surface water quality” and “pollution from other activities” were separately controlled, in three successive runs, to be with a 100% probability in a given state of interest (e.g. “low” or “high”). Results, presented in Figure 7, showed that water quality, itself partially driven by levels of external pollution, is expected to have a greater influence on biodiversity levels than the risk of spread of disease to native species.

#### 2.5 Resilience of local community

**Figure 8:**
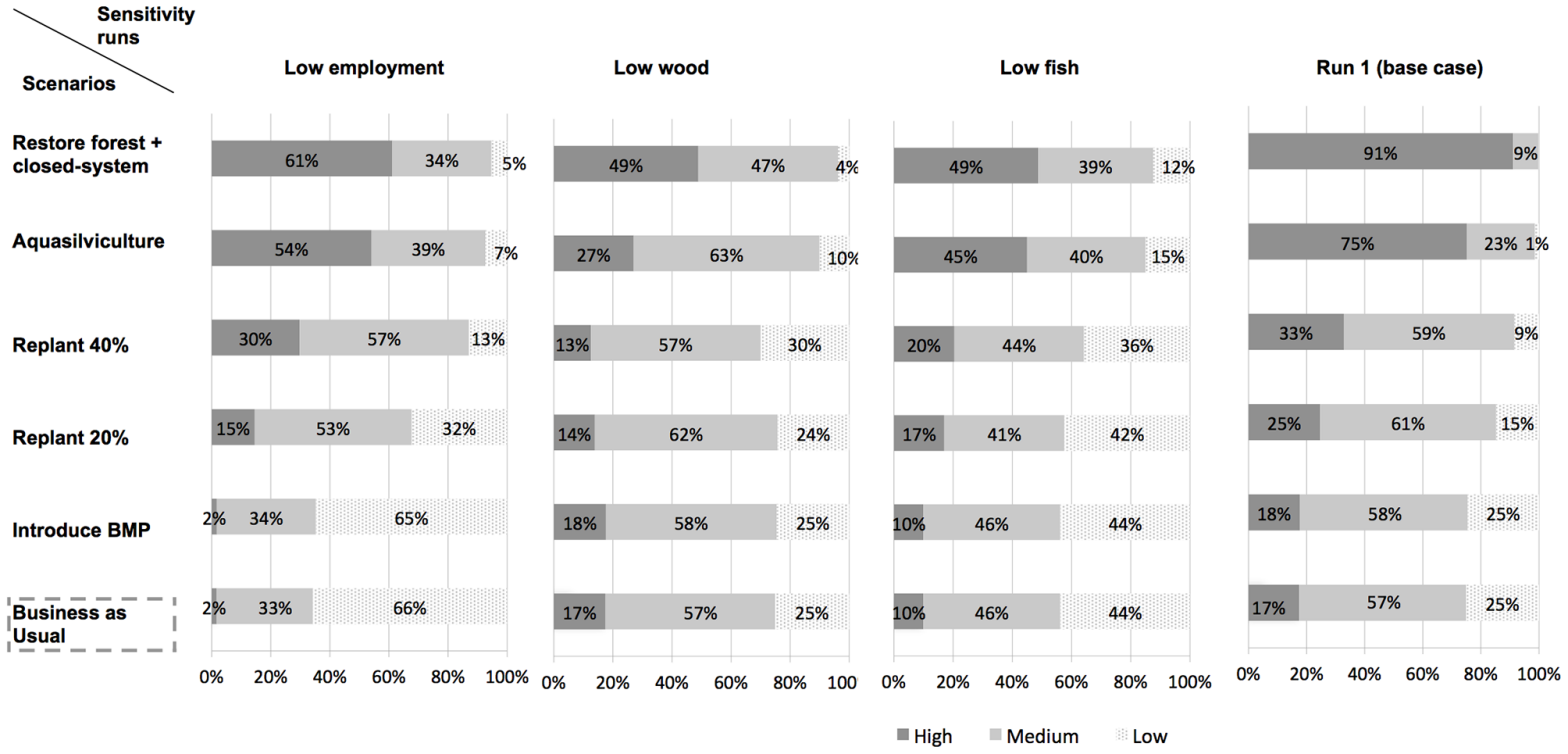
Sensitivity analyses for the node “Locals' subsistence capacity.” Assessment of the predictive influence of the variables “quantity of wood for locals”, “quantity of fish for locals” and “local employment” on the posterior probability distribution of the node “locals' subsistence capacity”.

Scenarios “restore forest+closed-system” and “aquasilviculture” stood out as very likely to bring high coastal community resilience, with a 93% and 82% probability respectively, vs. 27% under “BAU”. By contrast, the spread of posterior PDs under replanting scenarios showed much greater uncertainty in the expected level of resilience. Sensitivity analysis focused on the node “locals' subsistence capacity” as it strongly influenced the node “resilience of local community” (see Figure 5). Each parent node of the node “locals' subsistence capacity” (i.e. nodes “quantity of wood for locals”, “quantity of fish for locals” and “local employment”) was separately controlled, in three successive runs, to be in the state “low” with a 100% probability. Results against run 1, presented in Figure 8, showed that uncertainty in the level of restoration of productive services was the greatest contributor to overall uncertainty in subsistence capacity and thus, of resilience. Additionally, under the replanting and aquasilviculture scenarios, the likelihood of high subsistence capacity was found to decrease more under the “low wood” sensitivity run than under the “low fish” sensitivity run. However, when the mangrove forest was restored to its original state (scenario “restore forest+closed-system”), fish and wood provision were found to have an equal influence on locals' subsistence capacity.

## Discussion

### 1. Highlights and contextualisation of the modeling results

Firstly, economic results aim solely to provide an indication of the difference in scenarios' financial performance and of the economic impact of improving coastal protection by replanting mangroves. As such, they should not be used for predictive purposes.

Secondly, the differential performance of scenarios in terms of contribution to Thailand's shrimp exports underlines substantial differences in production capacity and reliability between systems. Indeed, the productive capacity of aquasilviculture is small compared to closed systems, which are themselves more reliable than conventional systems (“BAU”) due to better disease risk management. Additionally, results suggest that, in the long run, the loss in production capacity from mangroves replanting in ponds is at least partially offset by the increase in farm lifespan due to a lower bioaccumulation of pollutants.

Thirdly, the non-linearity in biodiversity provision highlighted in the results is of particular relevance for the identification of an optimal level of mangrove replanting. The impact of management scenarios on biodiversity is, however, expected to be more complex than our results suggest and should be validated by field data.

Fourthly, systems where farming is mixed with mangroves may differ in the way they control organisms entering and exiting the shrimp pond (M. Troell, personal communication, 2011). Therefore, when considering local community resilience, uncertainty about the level of restoration of productive services is likely to be larger for the provision of nursery habitat and fish seed than for wood. The greater uncertainty in the restoration of fish provision following mangrove replanting may explain why wood availability appears as a stronger contributor to locals' subsistence capacity than fish provision. This hypothesis should, however, be tested empirically.

### 2. Role of this BBN as a decision support tool for sustainable coastal resource use

The BBN presented here provided a holistic representation of the main user conflicts and trade-offs associated with various forms of shrimp farming development. Although not a predictive tool, this BBN is a means of articulating existing knowledge and beliefs about the multiple interactions of shrimp farming with the environment, the economy and livelihoods. It also enables to quantitatively compare the trade-offs associated with a range of aquaculture development strategies. In a context of renewed calls for the improved governance of the sector, especially in countries where the social-ecological resilience of coastal areas has been eroded in the past, this approach could help policy-makers understand the potential consequences of their decisions and increase the transparency of their policy choices by prompting them to explicitly value pre-defined sustainability criteria.

Sustainable land and farm management practices should be in line with the principles of the EAA and adaptable to local constraints as part of adaptive management [10]. Consequently, the objective of Thai policy-makers should not be to identify the best aquaculture scenario to promote to all coastal areas, but to define an appropriate diversified portfolio of environmentally-friendly and socially-acceptable practices, where aquasilviculture and closed-systems are only the extremes of a spectrum of possible options [11]. By making explicit how different alternatives may mediate the major trade-offs associated with shrimp farming, the BBN developed here can support the construction of such a diversified portfolio of management practices and inform policy choices.

Furthermore, since conventional intensive shrimp farmers have traditionally focussed on short-term profitability instead of sustainability [39], economic incentives are expected to play a key role in the successful diffusion and adoption of more sustainable management practices among aquaculturists. Potential governmental levers include the improvement of farmers' access to certified markets that drive a price premium, the set-up of microfinance schemes (e.g. to help fund capital-intensive closed-systems), and the implementation of schemes for payments for ecosystem services. Regarding the latter, and subject to property rights and farmers' acceptance, modeling findings on the estimation of the difference in financial performance between the “BAU” scenario and alternatives involving the restoration of mangroves ecosystem services could help in defining a level of compensation to provide to aquaculturists. However, as yields, costs and revenues can vary widely between sites [50], [51], our data on aquaculturists' profits would need to be complemented by other data on costs and revenues to generate reliable estimates of the impacts of management scenarios on aquaculturists' profits at local, regional or national scales [50], [51].

Although adjustments would be required, the model developed in this paper could be replicated for capturing trade-offs between aquaculture development and environmental and livelihood protection objectives in other countries of Southeast Asia (particularly the Philippines and Indonesia) and Latin America (e.g. Mexico, Honduras, Venezuela), where shrimp farming has displaced mangroves. The issue of scale, however, should be at the core of potential replications of the model. Local land and farm management practices should also be encompassed in wider integrated coastal assessments since “mangroves destruction goes beyond the shrimp industry alone” [52]. Additionally, in areas where shrimp pond abandonment has been a widespread phenomenon following disease outbreaks [5], the model could provide a basis for modeling the rehabilitation of disused shrimp ponds and restoration of mangrove ecosystem services. To transfer the application of the BBN to such cases, the extent to which the ecosystem has been altered, e.g. acidity levels, tidal hydrology and soil alteration, and the objective of ecosystem services restoration, e.g. coastline protection, supporting community livelihoods through restoration of coastal fisheries, or aquasilviculture development, should be incorporated [53]. Pond rehabilitation options for other commercial purposes such as salt production and coconut plantations could also be considered.

### 3. Suggestions for methodological improvements

Given the size of the network and for practical reasons, our elicitation exercise left what constituted “high”, “middle” and “low” levels for each variable open to experts' interpretation. Ideally, the discrete states of all variables should have been characterised by a wider consultation with them.

Furthermore, interactions with experts at each stage of the network development should be complemented with broader consultations with key stakeholders, such as aquaculturists, local communities, environmental organisations and government representatives. Not only this is likely to strengthen the robustness of the model [23], it should also ease the implementation of policy measures stemming from the modeling findings [46].

Finally, although it was decided to focus only on ecosystem services that had a substantial economic value or that could be considered in terms of their qualitative contribution to local communities' resilience, the BBN could be further refined by incorporating other ecosystem services such as cultural services and carbon sequestration. The integration of profits from the latter, potentially secured via payments for ecosystem services, could enable to more accurately model the financial performance associated with each land management scenario.

## Conclusion

This paper aimed to develop a Bayesian belief network (BBN) as a decision support system for mediating trade-offs between economic development, protection of natural ecosystems and coastal livelihoods, piloted in the case of the Thai coastal shrimp aquaculture. Modeling insights consisted of identifying for each land and aquaculture management scenario: (i) the expected magnitude of trade-offs due to user-conflicts and (ii) the level of uncertainty surrounding scenarios' performance on criteria reflecting stakeholders' diverse interests. Further analyses enabled quantitative measurement of the sensitivity of the model outputs to pre-defined assumptions (e.g. farm lifespan), input values (e.g. percentage of crop loss, pollution from external activities) and conditional probabilistic dependencies between the network's variables.

Whilst the BBN was developed for coastal shrimp farming in Thailand, suggestions were provided on how to apply this decision tool to other coastal aquaculture contexts. The presently developed BBN can therefore support the implementation of the Ecosystem Approach for Aquaculture in three ways: (i) by articulating available knowledge and beliefs on aquaculture's multiple interactions with the environment, the economy and livelihoods, (ii) by promoting comprehensiveness, explicit handling of uncertainty and transparency in the valuation of pre-defined sustainability criteria and (iii) by supporting innovative policy measures. Examples of such measures include the design of a diversified portfolio of sustainable farm management practices and of schemes of payments for ecosystem services. Finally, from a wider perspective, this research underlines the potential of BBNs to help frame the sustainable development of productive industries that interfere with the provision of ecosystem services.

## Supporting Information

Table S1 **Economic and production data used for profit computation (supporting information).**
(XLS)
